# New Appliances and Things Medical

**Published:** 1898-06-18

**Authors:** 


### NEW APPLIANCES AND THINGS MEDICAL.

[We shall be glad to receive, at our Office, 28 & 29, Southampton Street, Strand, London, W.C., from the manufacturers, specimens of all new preparations and appliances which may be brought out from time to time.]

### CONCENTRATED LEMONADE (EIFFEL TOWER).

(G. Foster Clark and Co., Eiffel Tower Factory, Maidstone.)

This concentrated lemonade, which is one of the most important of Fontayne's Eiffel Tower specialities, appears to us a highly practical and wholesome preparation for general use. From trial that we have made of it we are prepared to answer for the purity of the ingredients and the pleasantness of the flavour, retaining, as it does, the aroma and fragrance of the fresh fruit. For summer use a large stock of lemonade can be kept for domestic consumption ready for use at a moment's notice, and at a price which compares very favourably with lemonade prepared by the ordinary mode of expressing the juice from the fresh fruit. To prepare the beverage a packet of Eiffel Tower lemonade is emptied into a jug, a pound of sugar added and the whole dissolved in a pint of boiling water; a tablespoonful of the syrup thus produced added to a pint of cold water makes a delicious and refreshing drink. It may, of course, be used for flavouring jellies, for making lemon squashes, or various punches, &c.

### NEW CLINICAL THERMOMETER.

(S. Maw, Son, and Thompson, 7-12, Aldersgate Street, E.C.)

The novel feature of the clinical thermometer now being manufactured by the above firm consists in the ease with which the index may be shaken down after use. By a patented process the operation can be effected with the least possible trouble. By comparing it side by side with the ordinary thermometer the experimenter will be surprised at the difference in force required to effect the same object in the two varieties. Many a thermometer is broken in the act of shaking down the index, and for this reason, combined with the saving in energy and temper, Messrs. Maw's thermometer should appeal to those who have to take temperatures frequently. This new principle is applicable to all forms of clinical thermometers, and the difference in price between the old and new style, is only 8s. per dozen.

### OKOL ANTISEPTIC DRESSINGS.

(Sanitas Company, Limited, Bethnal Green, London, E.)

The Sanitas Company has recently forwarded us samples of their new antiseptic dressings—comprising lint, gauze, and absorbent wool; all of them are impregnated with Okol, an antiseptic of high germicidal properties. According to the analytical report of Dr. Samuel Rideal, Okol resembles certain other disinfectants now on the market, which are obtained by the destructive distillation of coal or other carbonaceous matter. The oily products resulting from this process, when properly treated and emulsified, constitute, as is well known, a most valuable series of antiseptics. For the most part they consist of hydrocarbon derivatives of the benzine series; these, in ordinary strength and in ordinary quantities, cannot be regarded as poisons when applied externally or internally to the human organism, but are very destructive to the life and growth of the lower animal and vegetable parasites; and in the case of Okol, one of the most satisfactory of this series of antiseptics, experiments have shown that a most deadly influence is exercised on the ordinary putrefactive and pathogenic bacteria. When present to the extent of 10 per cent. Okol is a most efficient germicide, although for ordinary purposes much stronger solution can safely be employed. Dressings impregnated with Okol may be regarded as of the greatest use in surgery, exercising no irritating action on the skin or tissues, and at the same time may be relied upon as germicides of proved efficiency.

